# Osmoregulation in zebrafish: ion transport mechanisms and functional regulation

**DOI:** 10.17179/excli2015-246

**Published:** 2015-05-11

**Authors:** Ying-Jey Guh, Chia-Hao Lin, Pung-Pung Hwang

**Affiliations:** 1 Institute of Cellular and Organismic Biology, Academia Sinica, Nakang, Taipei, Taiwan; 2 Institute of Biological Chemistry, Academia Sinica, Nakang, Taipei, Taiwan; 3 National Institute for Basic Biology, Myodaiji-cho, Okazaki, 444-8787, Japan

**Keywords:** Osmoregulation, ion regulation, ionocyte, hormone, zebrafish

## Abstract

Fish, like mammals, have to maintain their body fluid ionic and osmotic homeostasis through sophisticated iono-/osmoregulation mechanisms, which are conducted mainly by ionocytes of the gill (the skin in embryonic stages), instead of the renal tubular cells in mammals. Given the advantages in terms of genetic database availability and manipulation, zebrafish is an emerging model for research into regulatory and integrative physiology. At least five types of ionocytes, HR, NaR, NCC, SLC26, and KS cells, have been identified to carry out Na^+^ uptake/H^+^ secretion/NH_4_^+^ excretion, Ca^2+^ uptake, Na^+^/Cl^-^ uptake, K^+^ secretion, and Cl^-^ uptake/HCO_3_^-^ secretion, respectively, through distinct sets of transporters. Several hormones, namely isotocin, prolactin, cortisol, stanniocalcin-1, calcitonin, endothelin-1, vitamin D, parathyorid hormone 1, catecholamines, and the renin-angiotensin-system, have been demonstrated to positively or negatively regulate ion transport through specific receptors at different ionocytes stages, at either the transcriptional/translational or posttranslational level. The knowledge obtained using zebrafish answered many long-term contentious or unknown issues in the field of fish iono-/osmoregulation. The homology of ion transport pathways and hormone systems also means that the zebrafish model informs studies on mammals or other animal species, thereby providing insights into related fields.

## 1. Introduction

Fish, like other osmoregulatory vertebrates, have to maintain extracellular ionic and osmotic homeostasis for normal functioning of cellular activities and physiological processes. This is achieved through sophisticated transepithelial transport mechanisms, which are conserved from fish to human. However, unlike terrestrial mammals, fish face the challenging task of balancing the dramatic ionic/osmotic gradients between the aquatic environment and their body fluids. Furthermore, fish have to swiftly and accurately adjust their iono-/osmoregulation mechanisms to cope with the aquatic environment, which is generally fluctuant in terms of ionic composition and osmolality. An understanding of fish iono-/osmoregulation mechanisms not only enhances our knowledge of basic biology and comparative/evolutionary physiology, but is also relevant to fish aquaculture. In fish, the gills carry out the majority of iono-/osmoregulation mechanisms, whereas in mammals, similar tasks are performed by the kidneys (Evans et al., 2005[39]; Hwang and Lee, 2007[79]). Specified mitochondria-rich cells, ionocytes, are the major cells that express specific ion transporters (or enzymes), and thus are responsible for the transport of ions (mainly uptake of Na^+^, Cl^-^, and Ca^2+^, secretion of H^+^ or HCO_3_^-^, excretion of NH_4_^+^) in the gills of fresh water (FW)-acclimated fish, similar to mammalian renal tubular cells (Dymowska et al., 2012[33]; Evans, 2011[37]; Hwang et al., 2011[80]; Hwang and Lin, 2014[81]). Studies using traditional fish models (trout, killifish, eel, etc.) have established the basic and dogmatic principles of fish iono-/osmoregulation mechanisms; however, many contentious and unclear issues remain. This is probably because of the limitations in the research methodologies applied to traditional model species, highlighting the need for a more suitable model. Zebrafish, with its extensive genetic databases and applicability of molecular/cellular physiological approaches, is an emerging model in developmental biology and neurosciences, and has recently been used to study fish iono-/osmoregulation mechanisms; this new trend of using zebrafish as a model has enabled us to revise several long-term dogmas and, more importantly, provide new insights into related issues (Evans, 2011[37]; Hwang, 2009[77]; Hwang and Chou, 2013[78]; Hwang and Lin, 2014[81]; Hwang and Perry, 2010[82]; Kumai and Perry, 2012[103]; Kwong et al., 2014[107]). 

**Figure 1:**
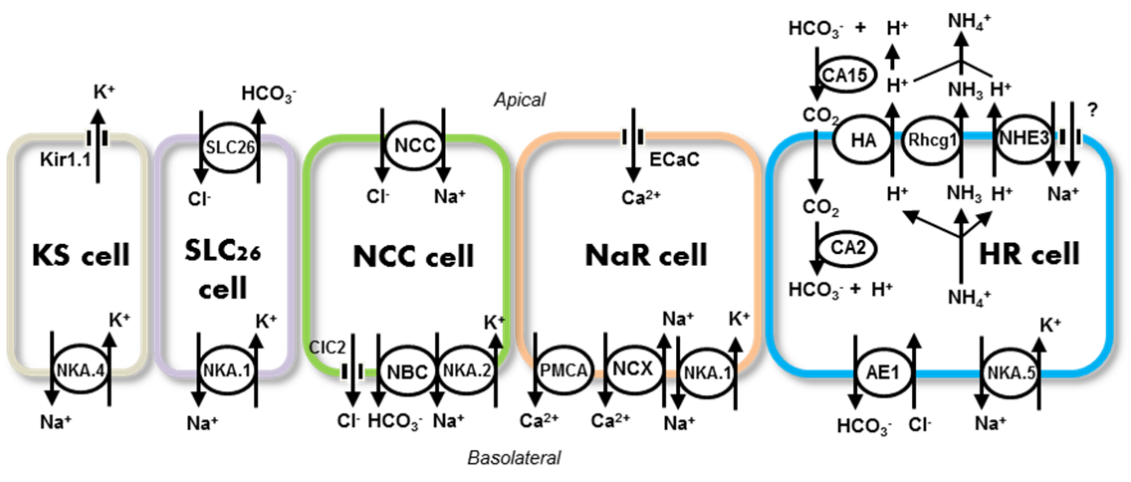
Ionocytes and ion transport pathways in zebrafish gills and skin. Five types of ionocytes have been identified: H+-ATPase-rich (HR), Na+-K+-ATPase-rich (NaR), Na+-Cl- cotransporter (NCC), solute carrier 26-expressing (SLC26), and K^+^ secreting (KS)-cells. Details of the transport pathways are given in the text (Sections 2-6). AE1, anion exchanger 1b; CA, carbonic anhydrase 2-like a; CA2 (-15), carbonic anhydrase 2-like a (-15a); ClC2, Cl- channel 2c; ECaC, epithelial Ca^2+^ channel; HA, H^+^-ATPase; ROMK, an ortholog of the mammalian renal outer medullary K+ channel (Kir1.1); NBC, electrogenic Na+-HO3- cotransporter 1b; NCC, Na+-Cl- cotransporter 2b; NCX, Na^+^/Ca^2+^ exchanger 1b; NHE3, Na^+^/H^+^ exchanger 3b; NAK.1~5, Na+-K+-ATPase α1 subunit subtypes (atp1a1a.1~5); PMCA, plasma membrane Ca^2+^-ATPase 2; Rhcg1, Rhesus glycoprotein; SLC26, SLC26A3, -4 and -6. Question marks (?) indicate unidentified transport pathways

In zebrafish, at least 5 types of ionocytes have been identified (Figure 1(Fig. 1)): (i) H^+^ATPase-rich (HR), (ii) Na^+^K^+^ATPase-rich (NaR), (iii) Na^+^-Cl^-^ cotransporter-expressing (NCC), (iv) solute carrier family 26 (SLC26)-expressing, and (v) K^+^secreting (KS) ionocytes; these cells express different sets of ion transporters, allowing them to conduct H^+^ secretion/Na^+^ uptake/NH4+ excretion, Ca^2+^ uptake, Na^+^/Cl^-^ uptake, K^+^ secretion, and Cl^-^ uptake/HCO_3_^-^ secretion, respectively (Hwang and Chou, 2013[78]; Hwang et al., 2011[80]; Hwang and Lin, 2014[81]). Ionocytes appear in the skin before the development of gill function, and therefore skin ionocytes substitute for those of the gills to perform iono-/osmoregulation during embryonic stages of fish. The ion transport pathways in zebrafish ionocytes have been summarized in various comprehensive fish reviews (Breves et al., 2014[12]; Dymowska et al., 2012[33]; Evans, 2008[38]; Evans, 2011[37]; Gilmour, 2012[49]; Hiroi and McCormick, 2012[64]; Hwang and Lee, 2007[79]; Hwang et al., 2011[80]; Kumai and Perry, 2012[104]; Wright and Wood, 2012[198]) and specific reviews on zebrafish (Chang and Hwang, 2011[19]; Hwang, 2009[77]; Hwang and Chou, 2013[78]; Hwang and Perry, 2010[82]; Kwong et al., 2014[107]). The present review is intended to introduce recent progress in the characterization of transport pathways of various ions and functional regulation by hormones. Particular efforts are made to compare zebrafish studies with studies on other fish species, to highlight how the former clarified long-term debates or unclear issues.

## 2. Na+ uptake, H+ secretion, and NH4+ excretion

### 2.1 Na^+^/H^+^ exchanger-mediated Na^+^ uptake

Several pathways have been proposed to be involved in fish Na^+^ uptake: electroneutral Na^+^/H^+^ exchange, H^+^-ATPase linking with epithelial Na^+^ channel (ENaC), and Na^+^-Cl^-^ cotransport. Na^+^ uptake coupled with acid secretion through electroneutral Na^+^/H^+^ exchangers (NHEs) has long been thought to occur in fish gill ionocytes, in a similar manner to mammalian renal tubular cells. Different isoforms of NHE (mainly, NHE2 and -3) have been identified, which differ with species (for details, please refer to the following reviews: Evans, 2011[37]; Hwang et al., 2011[80]; Kumai and Perry, 2012[104]). In zebrafish, as many as eight isoforms of NHE have been identified, and only one of them, NHE3b, has been demonstrated to be co-expressed with other transporters (for details, see below). Double or triple *in situ* hybridization/immunocytochemistry were used to reveal co-expression of H^+^-ATPase, carbonate anhydrases (apical CA15a and cytosolic CA2-like a), and anion exchanger (AE1b) in a specific group of ionocytes called HR cells (Lee et al., 2011[111]; Lin et al., 2006[121], 2008[122]; Yan et al., 2007[200]). The finding that NHE3 is the major isoform for apical Na^+^/H^+^ exchange in gill ionocytes was confirmed in many FW teleosts (dace, killifish, tilapia, medaka, eel, and others) (Hirata et al., 2003[62]; Hiroi et al., 2005[65]; Katoh et al., 2003[90]; Seo et al., 2013[155]; Wu et al., 2010[199]).

Most of the evidence for the role of NHEs in fish gill Na^+^ uptake is correlative or pharmacological (with NHE inhibitors) (for details, please refer to the following reviews: Evans et al., 2005[39]; Hwang, 2009[77]; Hwang and Perry, 2010[82]), and cannot distinguish between different NHE isoforms or be confined to NHE-expressing ionocytes. Zebrafish provided a platform to solve these issues. A fluorescence marker, sodium green, was used to specifically localize the signals of Na^+^ uptake in NHE3b-expressing HR ionocytes in zebrafish embryonic skin (Esaki et al., 2007[35]). Reinforcing the fluorescence evidence, non-invasive scanning ion-selective electrode technology (SIET) was used to precisely detect Na^+^ influx current in NHE3b-expressing ionocytes in the skin of intact medaka and zebrafish embryos (Lin et al., 2012[114]; Shih et al., 2012[160]; Wu et al., 2010[199]). Furthermore, the sodium green signals were suppressed either by incubation with NHE inhibitors or by inhibiting ionocyte differentiation through knockdown of Foxi3a (a transcription factor controlling ionocyte differentiation, see Section 7.5) (Esaki et al., 2007[35]); loss-of-function of NHE3b (through the use of specific morpholinos) resulted in impaired Na^+^ uptake or content in zebrafish embryo morphants (Chang et al., 2013[19]). These findings constitute solid molecular physiological evidence for the role of NHE3 in FW fish Na^+^ uptake.

### 2.2 H^+^-ATPase/ENaC-mediated Na^+^ uptake

Electric linking of transport through H^+^-ATPase with ENaC (originally identified in amphibian skin) has been suggested to take the place of electroneutral NHE in FW fish gill Na^+^ uptake mechanisms under unfavorable thermodynamic constraints on NHE (Avella and Bornancin, 1989[3]; Evans et al., 2005[39]; Kirschner, 2004[93]). Similar to studies using other species, pharmacological experiments in zebrafish provided major evidence in support of H^+^-ATPase/ENaC-mediated Na^+^ uptake in the gills. Treatment with bafilomycin (an H^+^-ATPase inhibitor) or amiloride (an ENaC inhibitor) resulted in impaired Na^+^ uptake, as shown by radioisotope tracing or sodium green fluorescence in zebrafish (Boisen et al., 2003[8]; Esaki et al., 2007[35]). Knockdown of H^+^-ATPase decreased Na^+^ content in zebrafish embryos, but only in low Na^+^ FW (Horng et al., 2007[70]). However, no ENaC genes could be found in the genomes of zebrafish and other teleost species (Venkatesh et al., 2007[181]), making the pathway a puzzle, at least in teleosts. This highlights the risk of misleading conclusions that are derived mainly from pharmacological or physiological experiments without the support of molecular evidence. 

Recently, acid-sensitive ion channels (ASICs), the closest relatives to ENaC, were identified; functional analysis of ASICs revealed that they are putative Na^+^ channels involved in apical Na^+ ^transport in FW fish gill cells. ASIC protein signals (detected using an anti-zebrafish ASIC4.2 antibody) were found to be co-localized with Na^+^-K^+^-ATPase in a group of gill ionocytes in FW rainbow trout, and treatment with ASIC inhibitors (4',6-diamidino-2-phenylindole and diminazene) suppressed Na^+^ influx in the gills (as shown by radioisotope tracing) (Dymowska et al., 2014[34]). This long-sought after evidence for the H^+^-ATPase-linked Na^+^ channel pathway is plausible. However, more findings are required to support this hypothesis; for example, more precise mRNA localization of specific ASIC isoform(s), detection of co-localization with apical H^+^-ATPase, and investigations using other species.

### 2.3 H^+^ secretion

H^+^-ATPase and NHE are two pathways responsible for acid secretion in fish gills. One of the primary evidences for the acid secretion function of apical H^+^-ATPase and NHEs is the effect of environmental acid (or high CO_2_) or internal acidosis on gene expression or acid secretion in the gills (for details, please refer to the following reviews: Evans et al., 2005[39]; Hwang and Lin, 2014[81]). Previous studies were unable to localize H^+^ efflux currents in fish gill cells, or monitor such currents in real-time; such experiments became feasible with the advent of SIET, which enabled detection of ionic currents in the embryonic skin of intact zebrafish (as well as tilapia and medaka) (Horng et al., 2009[69]; Lin et al., 2006[121]; Wu et al., 2010[199]). SIET was combined with gene knockdown and pharmacological approaches to provide the first convincing molecular physiological evidence that H^+^-ATPase- and NHE3b-mediated proton secretion function precisely at the apex of HR ionocytes in zebrafish (Shih et al., 2012[160]). Subsequent detailed studies further discriminated between the contributions of H^+^-ATPase and NHE3b in acid secretion: H^+^-ATPase, plays a greater role, with 67-75%, while NHE3b contributes 27-29% of the apical H^+^ activity in HR cells (Shih et al., 2008[158], 2012[160]). On the other hand, medaka, like most FW teleosts, adopts NHE3 as the sole apical transporter to secret acid (Lin et al., 2012[121]), with H^+^ activity in NHE3-expressing ionocytes being 27-fold that of other cells in the embryonic skin (Lin et al., 2012a[121]). It appears that acid secretion largely depends on apical H^+^-ATPase in stenohaline zebrafish, but apical NHE3 in euryhaline medaka (and most other species). This trend is of evolutionary physiological significance, and needs to be clarified in more species.

### 2.4 NH_4_^+^ excretion coupled with NHE and/or H^+^-ATPase 

Most teleosts are ammonotelic, and mainly excrete ammonia as waste nitrogen from the gills (or the skin during embryonic stages); this is in contrast to mammals, which produce less toxic urea through energy-consuming conversion, and excrete it through the kidneys (Evans et al., 2005[39]; Hwang et al., 2011[80]). The physiology of ammonia excretion in fish has long been investigated, but the detailed molecular mechanisms were not unraveled until the identification of Rhesus (Rh) glycoproteins, a group of ammonia transporters. Nakada et al. (2007[135]) first identified ammonia transporters (Rhag, -bg, -cg1, and -cg2) in gill cells of pufferfish, and then further reported restriction of Rhcg1 to a specific group of acid-secreting ionocytes, HR cells, in zebrafish (Nakada et al., 2007[134]). This opened a window to the study of ammonia transport mechanisms associated with H+ secretion and Na+ uptake in FW fish gills. 

In zebrafish, HR ionocytes express apical Rhcg1 (Nakada et al., 2007[134]; Shih et al., 2008[158]), while keratinocytes (i.e., pavement cells) express both apical and basolateral Rhbg (Shih et al., 2013[159]). Rhcg1 and other isoforms have been also identified in many species, and the functional roles of these transporters were proposed mainly on the basis of the correlation between transporter expression levels and ammonia excretion rates and/or the effects of environmental ammonia concentrations on these parameters (for details, please refer to the following reviews: Evans, 2011[37]; Hwang et al., 2011[80]; Hwang and Lin, 2014[81]; Wright and Wood, 2009[198], 2012[198]). Because of the lack of inhibitors of Rh proteins, loss-of-function combined with SIET in zebrafish was used to provide convincing and direct molecular physiological evidence that Rhcg1 is the major player in ammonia excretion; for the first time, NH_4_^+^ and H^+^ currents were specifically detected in real-time at the apex of HR cells, and were impaired by knockdown of Rhcg1 and H^+^-ATPase, respectively (Shih et al., 2008[158]). Other Rh isoforms, -ag and -bg, were also reported to be expressed in cell types other than HR ionocytes in zebrafish skin, and knockdown of each of these isoforms resulted in similar levels of impaired ammonia excretion, suggesting that these isoforms have similar roles, without interacting with one another (Braun et al., 2009[11]). A recent study used sophisticated triple labelling and cell-level SIET to demonstrate that Rhcg1 and -bg show distinct expression patterns and subtly different transport capacities in the zebrafish ammonia excretion mechanism (Shih et al., 2013[159]). In contrast to Rhcg1 expression in the apical membrane of HR ionocytes, Rhbg is expressed only in keratinocytes at both the apical and basolateral regions. Rhcg1 and Rhbg are responsible for NH_4_^+^ secretion in HR cells and keratinocytes, respectively, as determined by loss-of-function experiments. Further detailed SIET analyses indicated that Rhbg-expressing keratinocytes with a larger surface area mainly contribute to basal ammonia excretion in normal situations (Shih et al., 2008[158]), while Rhcg1-expressing HR ionocytes with a high transport capacity are the major contributor under harsh conditions, like high ammonia (Shih et al., 2013[159]). These differential expression levels and functional capacities of ammonia transporters between the two cell types is of physiological significance, as it enables zebrafish to efficiently cope with different environments. Rhcg1 is also the major isoform present in a group of NHE3-expressing ionocytes, but not keratinocytes, of medaka, as discovered using similar multiple labeling approaches and SIET (Lin et al., 2012[114]; Wu et al., 2010[199]). On the other hand, in FW trout, pavement cells (keratinocytes), rather than mitochondria-rich cells (ionocytes), have been proposed to be the major player for ammonia excretion in a high ammonia environment, and this pathway is mainly mediated by Rhcg2, but not Rhcg1, according to studies of isolated gill cells and *in vivo* gill experiments without cellular localization data (Nawata et al., 2007[136]; Wood and Nawata, 2011[195]). This, Rhcg1 in zebrafish (and medaka) vs Rhcg2 in trout, may be considered as a species-dependent difference. However, a recent study using delicate double immunocytochemistry with homologous antibodies demonstrated co-localization of apical Rhcg1 and NHE3 in a specific group of ionocytes in trout gills (Takei et al., 2014[169]). Fish gills are an organ with various types of cells, and each cell type may express distinct isoform(s) of a gene family. As such, the conclusions derived merely from physiological or molecular experiments in fish gills should be carefully re-evaluated if the findings are not supported by precise localization and/or identification of the target genes in each cell type.

### 2.5 NH_4_^+^-dependent Na^+^ uptake mechanism: driving force for NHE

Unlike mammalian proximal tubular cells, fish gill ionocytes face a hypoosmotic FW environment, which results in Na^+^ and H^+^ gradients that are thermodynamically unfavorable to the operation of NHE; this situation has long been an issue of some debates in the studies of the physiology of fish inhabiting FW (Avella and Bornancin, 1989[3]; Parks et al., 2008[139]). The recent discovery of coupled Rhcg1 and NHE3 and the related SIET analyses enabled us to resolve the debates by presenting an ammonia-dependent Na^+^ uptake mechanism. Krogh (1939[96]) initially found that ammonia excretion and Na^+^ uptake are linked in FW fish. Wright et al. (1989[196]) proposed an acid-trapping hypothesis of ammonia excretion in FW gills: excreted CO_2_ results in an acidified external boundary layer that converts apical NH_3_ to NH_4_^+^, and thus facilitates the diffusion of NH_3_ through the apical membrane of gill cells. Following the discovery of Rh proteins in fish gills, Rhcg1 was identified as the major isoform co-expressed with H^+^-ATPase and/or NHE3 in the ionocytes of zebrafish and medaka (Nakada et al., 2007[134][135]; Shih et al., 2008[158]; Wu et al., 2010[199]). SIET analyses combined with morpholino knockdown and/or pharmacological experiments resulted in convincing real-time evidence for an alternative acid-trapping-mediated ammonia excretion mechanism, in which apical H^+^ is derived mainly from the operation of H^+^-ATPase and/or NHE3 in zebrafish (Shih et al., 2008[158]) and medaka (Wu et al., 2010[199]). Integrating the data from trout and other species, (Wright and Wood, 2009[197]) further proposed a plausible and attractive model of an “Na^+^/NH_4_^+^ exchange complex” consisting of several membrane transporters (Rhcg, H^+^-ATPase, NHE2/-3, ENaC) working together as a metabolon; this metabolon provides an acid trapping mechanism for apical ammonia excretion, with external CO_2_ as the major source of H^+^. However, there are some uncertainties regarding the transporter isoforms (Rhcg1 or -2, NHE3 or -2) and the gill cell types (ionocytes or pavement cells). In medaka embryonic skin, real-time currents of NH_4_^+^excretion, Na^+^ uptake, and H^+^ secretion were detected in the same ionocytes, and they are tightly linked according to SIET analyses (Wu et al., 2010[199]); high-NH_4_^+^ acclimation induced compensatory NH_4_^+^excretion and increased Na^+^ uptake, while an acute high-NH_4_^+^ medium blocked NH_4_^+^excretion with a concomitant decrease in Na^+^ uptake. These findings provided more precise evidence to support and further refine Wright and Wood's model (Wright and Wood, 2009[197]). NHE3-mediated Na^+^ uptake in FW fish gill ionocytes is proposed to involve Rhcg1-mediated deprotonation of NH_4_^+^ inside gill ionocytes, which facilitates NH_3_ gas diffusion down the gradient generated by the acid trapping of NH_3_ outside the membrane; as such, NH_4_^+^ excretion may generate an H^+^ gradient across the apical membrane to drive Na^+^ uptake through NHE3 (Wu et al., 2010[199]). The hypothesis that an NH_4_^+^ excretion-created H^+^ gradient drives electroneutral NHE3 was further supported by evidence from studies of fish under harsh environments. Knockdown of Rhcg1 or acute NH_4_^+^ treatment resulted in simultaneous suppression of both NH_4_^+^ excretion and Na^+^ uptake in zebrafish embryos acclimated to low-Na^+^ (Shih et al., 2012[160]) or low-pH FW (Kumai and Perry, 2011[104]). Moreover, these harsh environments stimulated NH_4_^+ ^excretion in ionocytes and gill mRNA expression of Rhcg1 in zebrafish (Shih et al., 2008[158], 2012[160]) and medaka (Lin et al., 2012[121]; Wu et al., 2010[199]). The presence of the NH_4_^+^-dependent Na^+^ uptake machinery may reasonably be predicted to provide the driving pathway for electroneutral Na^+^/H^+^ exchange via NHEs in gill ionocytes of FW fish (Figure 1(Fig. 1)), and probably also in other FW organisms. In the words of Hirose and Nakada (Hirose and Nakada, 2010[67]), the SIET study in medaka by Wu et al. (2010[199]) “…added a further twist to this adaptive strategy by providing evidence for a coupling of the ammonia excretion with a sodium uptake process, which is essential for living in freshwater conditions”.

### 2.6 Carbonate anhydrases and basolateral transporters 

Apical NHE3- and/or H^+^-ATPase-mediated Na^+^ uptake and H^+^ secretion (equivalent to HCO_3_^-^ uptake) in zebrafish HR cells or other fish ionocytes (e.g. trout PNa^+^ cells, tilapia type III cells, medaka NHE3-expressing cells) is analogous to mammalian kidney proximal tubules and collecting ducts (Evans et al., 2005[39]; Hwang et al., 2011[80]), and requires carbonate anhydrases (CAs) and other basolateral transporters to achieve the transepithelial transport function; however, the precise localization data and functional analyses for these enzymes and transporters were mostly obtained from zebrafish. Cytosolic CA (CA2) has been identified and well-documented, while the membrane form of CA (CA4 in mammals) was not detected in fish gill ionocytes until the recent zebrafish studies (Gilmour, 2012[49]; Gilmour and Perry, 2009[50]; Hwang, 2009[77]; Lin et al., 2008[122]). CA15a (an ortholog of mammalian CA4) and CA2 like-a (an ortholog of mammalian CA2) were identified to be co-expressed in zebrafish HR ionocytes, and loss-of-function experiments indicated that these proteins participate in NHE3/H^+^-ATPase-mediated Na^+^ uptake and H^+^ secretion mechanisms (Lin et al., 2008[122]). The most likely candidate for the basolateral transporter is Na^+^-HCO_3_^-^ cotransporter (NBCe1), as it has a functional association with acid-base regulation and Na^+^ uptake in FW fish gills (Evans et al., 2005[39]). A landmark study of acid-tolerant dace provided comprehensive and convincing data for the role of NBC (Hirata et al., 2003[62]), as follows:

(i) localization of NBC in Na^+^-K^+^-ATPase-labeled gill ionocytes, 

(ii) functional analysis of NBC in an oocyte overexpression system, and 

(iii) observed enhancement of NBC mRNA expression after acid acclimation.

This putative role of NBC was subsequently reaffirmed in different species with correlated molecular, physiological, and pharmacological data (for details, please refer to the following reviews: Evans, 2011[37]; Evans et al., 2005[39]; Hwang and Lee, 2007[79]), despite a lack of evidence for co-localization of NBC with NHEs or H^+^-ATPase in specific fish gill ionocytes. A recent study in zebrafish revised this dogma (Lee et al., 2011[111]). Among 14 isoforms of the SLC4 family, only SLC4A1b (AE1b) and -4A4b (NBCe1b) exhibit a salt-and-pepper ionocyte pattern following *in situ* hybridization. Furthermore, AE1b and NBCe1b respectively co-localize in the HR and NCC types of ionocytes in zebrafish; basolateral AE1b, but not NBCe1b, were identified as the major transporter for acid secretion/Na^+^ uptake mechanisms in collaboration with apical H^+^-ATPase/NHE3b and the two CAs (-2like a and -15a) in HR cells, based on loss-of-function and SIET experiments (Lee et al., 2011[111]). This new insight was supported by subsequent co-localization studies in tilapia and medaka, which showed that NBCe1 is specifically expressed in NCC-expressing ionocytes, but not in cells expressing NHE3 (Furukawa et al., 2011[44]; Hsu et al., 2014[75]). The conclusions of the previous studies addressing the role of NBC in fish gill acid-base regulation and/or Na^+^ uptake functions await further re-clarification in the future. 

The zebrafish model for H^+^ secretion/Na^+^ uptake mechanisms (Figure 1(Fig. 1)) was established through serial molecular physiological studies combining double/triple *in situ* hybridization/immunocytochemistry, knockdown/overexpression, and SIET approaches (Hwang and Chou, 2013[78]; Hwang et al., 2011[80]; Hwang and Lin, 2014[81]). In HR ionocytes, apical NHE3b and H^+^-ATPase transport H^+^ out of cells, and H^+^ combines with environmental HCO_3_^-^ to generate H_2_O and CO_2_, as catalyzed by CA15a (an ortholog of mammalian CA4). Carbon dioxide enters cells and is hydrated by CA2 like-a (an ortholog of mammalian CA2) to form H^+^ and HCO_3_^-^. Basolateral AE1b (an ortholog of mammalian AE1) extrudes cytosolic HCO_3_^- ^down the Cl^-^ gradient to fulfill the epithelial acid-secreting function, while basolateral Na^+^-K^+^-ATPase (Liao et al., 2009[112]) is responsible for the excretion of Na^+^. The observed transport mechanisms in zebrafish HR ionocytes were further supported by a recent sensitive study, in which a proximity ligation assay was adopted to demonstrate that NHE3b, Rhcg1, CA15a, and CA2-like a form and function as a transport metabolon in HR cells (Yusuke et al., 2012[203]). Some publications favor the action of membrane CA (e.g. CA15a or CA4) on CO_2_ hydration, based on the very low level of HCO_3_^-^ in FW (Gilmour, 2012[49]; Wright and Wood, 2009[197]). In an early study using rainbow trout, the HCO_3_^-^ concentration in the external water was found to fall as FW media flowed pass the gills, and this event was suppressed by acetazolamide (a CA inhibitor), but not affected by SITS (an anion exchanger inhibitor) (Lin and Randall, 1991[119]), suggesting that secreted H^+^ results in dehydration of HCO_3_^-^ (not CO_2_ hydration) in the unstirred layer (Evans et al., 2005[39]). As such, dehydration of HCO_3_^-^ occurs at the apical surface of gill cells, and requires acidification by H^+^. Moreover, knockdown of CA15a resulted in prompt acidification at the apical membrane of HR cells in zebrafish (Lin et al., 2008[122]), convincingly demonstrating the action of CA15a on HCO_3_^-^ dehydration, and thus supporting the proposed transport machinery of HR cells.

## 3. NCC-mediated Na+ and Cl- uptake

In addition to ionocytes expressing NHEs, NCC-expressing cells have recently been suggested to be another main type of ionocyte in FW fish gills (Figure 1(Fig. 1)) (Evans 2011[37]; Hwang et al. 2011[80]; Dymowska et al. 2012[33]; Hiroi and McCormick, 2012[64]; Hwang and Lin, 2014[81]). Staining with a heterologous antibody (T4) resulted in the initial identification of apical NKCC in a subset of ionocytes distinguishable from the NHE3-expressing cells in tilapia gills and embryonic skin (Hiroi et al., 2005[65]). However, this finding was later revised by the same group, who were the first to establish that NCC is a novel transporter involved in Na^+^ and Cl^-^ uptake in FW fish gills, as shown by the use of delicate and convincing multiple-label immunocytochemistry (Hiroi et al., 2008[66]; Inokuchi et al., 2009[84], 2008[85]). Gill-specific NCC (SLC12A10), distinct from the kidney-dominant NCC (SLC12A3), is a fish-specific isoform of the SLC12A family (Hiroi et al., 2008[66]; Wang et al., 2009[187]), and was recently named NCC2 (Hartmann et al., 2014[57]; Takei et al., 2014[169]). In tilapia, NCC2 is the only NKCC/NCC isoform to be expressed at the apex of FW-type ionocytes; moreover, its expression (at the levels of mRNA expression and cell number) is stimulated by FW, deionized FW, or low-Cl^-^ FW (Hiroi et al., 2008[66]; Inokuchi et al., 2009[84], 2008[85]). Furthermore, SIET detected Cl^-^ influx currents only in the NCC-expressing ionocytes of tilapia embryonic skin; such current was stimulated after acclimation to low-Cl^-^ FW and suppressed up to 90% by metolazone, an NCC inhibitor (Horng et al., 2009[69]). A study in zebrafish reinforced those novel findings from tilapia (Wang et al., 2009[187]). NCC2b (formerly NCC-like 2) was found to be expressed in a specific type of ionocyte distinct from HR and NaR cells, and stimulated in low-Cl^-^ FW; NCC2b loss-of-function by morpholino knockdown impaired both Cl^-^ influx and content in the embryos. The role of NCC2 in Cl^-^ uptake function in FW gills has been better elucidated than its role in Na^+^ uptake (Wang et al., 2009[187]); it is, however, known that metolazone treatment suppresses Na^+^ influx, but NCC2b knockdown increases Na^+^ content with a concomitant enhancement of NHE3b expression in zebrafish embryos (Wang et al., 2009[187]). A recent study provided information with physiological and evolutionary significance. Low-pH FW was reported to suppress both NHE3 expression in HR ionocytes and Na^+^ uptake function (Chang et al., 2013[19]; Yan et al., 2007[200]), and this was followed by a compensatory increase in Na^+^ influx (Kumai et al., 2011[98]). Compensatory Na^+^ uptake is mainly derived from enhanced NCC2b mRNA expression/ionocyte number under acidic stress (Chang et al., 2013[19]). In support of this model, knockdown of NHE3b or Gcm2 (a transcription factor controlling the differentiation of HR ionocytes, see Section 7.5) resulted in an increase of NCC cell number with a concomitant recovery of Na^+^ uptake function (Chang et al., 2013[19]). Taken together, these findings indicate that NCC2 in NCC-expressing ionocytes plays a functionally redundant role in Na^+^ uptake mechanisms in zebrafish under harsh environments that disturb body fluid Na^+^ homeostasis, and the functional redundancy and mutual compensation between NHE3 and NCC2 in Na^+^ absorption appear to be conserved in zebrafish gill/skin ionocytes (HR vs NCC cells) and mammalian kidney cells (the proximal vs. distal convoluted tubular cells) (Chang et al., 2013[19]). 

**Figure 2:**
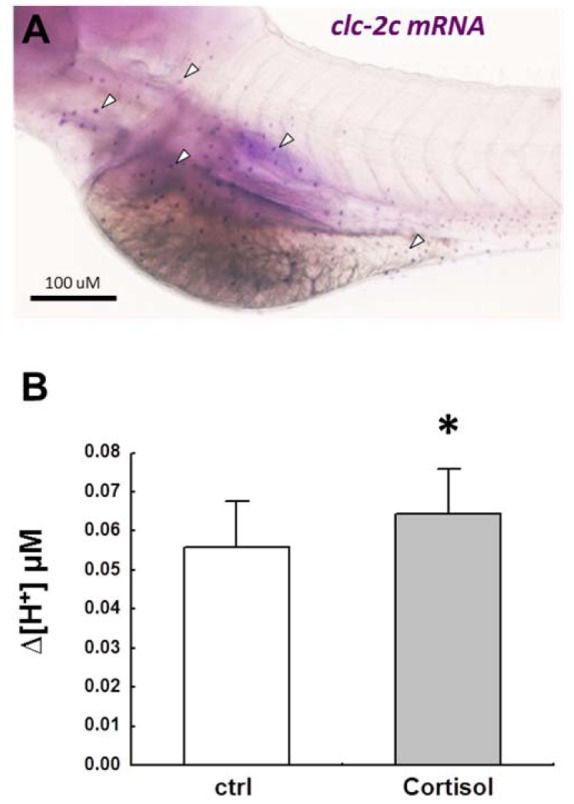
(A): *In situ* hybridization of *clc-2c* in zebrafish at 5 d-post-fertilization. The mRNA signas (arrow head) of *clc-2c* were expressed in a pattern similar to that of ionocytes. RNA probe for *in situ* hybridization: *zclc-2c* (ENSDARG00000060439), nt598-1705. (B) Exogenous cortisol (20 mg/mL) stimulated H^+^ secretion (as detected by SIET) in the skin of 3 d-post-fertilization zebrafish. *, Significant difference from control group (P < 0.05, Student's *t*-test).

CLC chloride channels, NBCe1, and Na^+^-K^+^-ATPase were recently demonstrated to be the major basolateral transporters for NCC-mediated ransepithelial Na^+^/Cl^-^ uptake (Figure 1(Fig. 1)). CLC-3 is localized at the basolateral membranes of a specific group of ionocytes (Na^+^-K^+^-ATPase- or NCC2b-labeled) in several species other than zebrafish, and protein expression of CLC-3 is up-regulated after acclimation to FW or low-Cl^-^ FW (although there are conflicting results at the mRNA level) (Bossus et al., 2013[9]; Tang et al., 2010[173]; Tang and Lee, 2007[175], 2011[174]). On the other hand, in our recent preliminary study of zebrafish, we used *in situ* hybridization (Figure 2A(Fig. 2)) combined with homologous antibody immunocytochemistry to reveal that CLC-2c, but not CLC-3, is co-expressed with NCC2b in the same ionocytes, and found that CLC-2c knockdown decreased Cl^-^ content (Wang et al., unpublished data). CLC-2 or -3 may be a species-dependent difference; however, one could not exclude a possible risk of misidentifying the target transporter from data using a heterologous antibody in the absence of convincing support from *in situ* hybridization data. 

As mentioned above, NBCe1 is co-expressed with NCC2 in the same types of ionocyte in tilapia (Furukawa et al., 2011[44]) and zebrafish (Lee et al., 2011[111]); however, the functional role of NBCe1 in ionic regulation mechanisms in these species are not yet clear. In tilapia, acidic (pH 4) FW did not affect NBCe1 mRNA expression, or the cell density and size of NCC-expressing ionocytes in the gills of tilapia; furthermore, FW supplemented with 50 mM NaCl showed a (non-significant) trend toward suppressing NBC expression (Furukawa et al., 2011[44]). In the case of zebrafish, NBCe1b expression was suppressed after acclimation to pH-4 or low-Na^+^ FW, while expression of AE1b (a transporter involved in acid-secretion and Na^+^ uptake) was stimulated by the same treatment ((Lee et al., 2011[111]); refer to Section 2.6). The exact role of NBC in NCC-expressing ionocytes remains to be explored.

## 4. Solute carrier 26-mediated Cl- uptake and HCO3- secretion

Chloride ion uptake coupled with HCO_3_^-^ extrusion was first proposed to be a pathway for facilitating gas exchange in fish gills (Krogh, 1937[97]), and was thereafter considered a mechanism for ionic and acid-base regulation through Cl^-^/HCO_3_^-^ exchangers (for details, please refer to the following review: Evans, 2011[37]). Initially, AE was identified as the transporter for apical Cl^-^/HCO_3_^-^ exchange in the gills of several FW species, with such identification being mainly based on pharmacological/physiological experiments and immunocytochemistry with heterologous antibodies (Chang and Hwang, 2004[16]; Perry and Randall, 1981[143]; Preest et al., 2005[148]; Sullivan et al., 1996[163]; Tang and Lee, 2007[175]; Wilson et al., 2000[193]). AE1b was recently found to function as a basolateral transporter for acid secretion and/or Na^+^ uptake in zebrafish HR ionocytes (Lee et al., 2011[111]) (see Section 2.6 above), thereby rendering the identity of the apical Cl^-^/HCO_3_^-^ exchanger in fish gills a mystery. A study of the euryhaline stingray by Piermarini et al. (Piermarini et al., 2002[145]) identified pendrin, a solute carrier 26 member (SLC26A4), as another candidate for apical Cl^-^/HCO_3_^-^ exchange, thus providing some clues towards resolving this issue. Zebrafish was used as an alternative and competent model to provide convincing evidence for the identification and functional characterization of SLC26s in FW gill Cl^-^ uptake and HCO_3_^-^ secretion mechanisms (Bayaa et al., 2009[6]; Perry et al., 2009[144]). Three orthologs of mammalian pendrin, SLC26A3, -4, and -6, were identified and located at the mRNA level in certain type of cell in the embryonic and adult gills, and some SLC26A3 signals were co-localized in Na^+^-K^+^-ATPase rich (NaR) ionocytes (Bayaa et al., 2009[6]; Perry et al., 2009[144]). The mRNA expression of the SLC26 members and Cl^-^ uptake in the gills were stimulated after acclimation to low-Cl^-^ or high-NaHCO_3_ FW, suggesting the SLC26 members play roles in in apical Cl^-^/HCO_3_^-^ exchange in zebrafish gill cells (Perry et al., 2009[144]). This hypothesis was further reinforced by loss-of-function experiments, in which knockdown of SLC26A3, -4, and -6 respectively resulted in impaired Cl^-^ uptake in morphants raised in low-Cl^-^ FW (Bayaa et al., 2009[6]). A recent study in rainbow trout also supported the role of SLC26A6 in Cl^-^ uptake in FW gills (Boyle et al., 2015[10]); the gene was predominantly expressed in the gills, and Cl^-^ influx was suppressed by ambient CO_2_ and some inhibitors (acetazolamide and DIDS). According to the model of type B intercalated cells of the mammal collecting duct (Wagner et al., 2009[182]), zebrafish SLC26s-expressing cells have to co-express CAs (membrane and cytosolic forms), basolateral Cl^-^ channels, and H^+^-ATPase to achieve transepithelial Cl^-^ uptake and HCO_3_^-^ secretion in the gill or skin. The search for evidence of co-localization and co-functioning of these enzymes/transporters with SLC26s in the same ionocytes continues, although some pharmacological studies suggest roles for CA and H^+^-ATPase in gill Cl^-^ uptake in certain FW fish, including zebrafish, tilapia, and trout (Boisen et al., 2003[8]; Boyle et al., 2015[10]; Chang and Hwang, 2004[16]).

## 5. Ca2+ uptake

While some Ca^2+^ is obtained from the diet, necessary Ca^2+^ is mainly absorbed from the aquatic environment by the gills to maintain body fluid Ca^2+^ homeostasis in FW fish (Flik et al., 1995[43]). The current model of mammalian transcellular Ca^2+^ transport (Hoenderop et al., 2005[68]) was proposed to be conserved in FW fish gills based on early physiological and biochemical studies (Flik et al., 1995[43]): Ca^2+^ is transported through apical epithelial Ca^2+^ channels (ECaC) into gill ionocytes, and is then extruded via the basolateral plasma membrane Ca^2+^-ATPase (PMCA) and Na^+^/Ca^2+^ exchanger (NCX), while the operation of NCX is driven by the Na^+^ gradient created by Na^+^-K^+^-ATPase. However, the identities and the functional properties of these Ca^2+^ transporters were unclear until recent molecular physiological studies. Fish ECaC was initially cloned from fugu (Qiu and Hogstrand, 2004[149]), and thereafter from other species, including zebrafish (Hsu et al., 2014[75]; Pan et al., 2005[138]; Shahsavarani et al., 2006[156]; Takabe et al., 2012[167]). By exploiting the advantages provided by plentiful genetic databases, serial studies in zebrafish have significantly advanced our knowledge of the molecular physiology of fish gill Ca^2+^ uptake. Only one PMCA member (out of six), PMCA2, one NCX member (out of seven), NCX1b, and one Na^+^-K^+^-ATPase subunit (out of six), ATP1A1a.2, were identified to be co-expressed with ECaC in the same type of NaR ionocytes in zebrafish gill and embryonic skin (Liao et al., 2007[113]; Pan et al., 2005[138]). Low-Ca^2+^ FW acclimation stimulated both ECaC mRNA expression and increased ECaC-expressing ionocyte number in zebrafish embryonic skin (Pan et al., 2005[138]); ECaC loss-of-function through morpholino knockdown resulted in impaired Ca^2+^ uptake and Ca^2+^ balance in zebrafish morphants, demonstrating the role of ECaC in FW fish body fluid Ca^2+^ homeostasis (Tseng et al., 2009[178]). Supporting the findings in zebrafish, a study in rainbow trout also reported stimulation of ECaC expression in the gills upon soft water acclimation or CaCl_2_ infusion (Shahsavarani et al., 2006[156]). In zebrafish gills, only the mRNA expression of ECaC, but not PMCA1 nor NCX1b, responds to environmental Ca^2+^ levels (Liao et al., 2007[113]) or hormonal treatments (please refer to Section 7.2). Such evidence supports the functional role of ECaC as a rate-limiting step or a major regulatory transporter of FW fish gill Ca^2+^ uptake, as also observed for mammalian kidneys (Flik et al., 1997[40]; Hoenderop et al., 2005[68]). Interestingly, trout ECaC is expressed in both pavement cells and ionocytes in the gills (Shahsavarani et al., 2006[156]), while in medaka, detailed triple *in situ* hybridization/immunocytochemistry analyses revealed that ECaC is specifically expressed in a group of ionocytes (presumably accessory cells), which are always adjacent to other types of NHE-expressing ionocyte (Hsu et al., 2014[75]). As such, the zebrafish studies established the basic molecular physiological model of ECaC-mediated Ca^2+^ uptake; however, this mechanism is worthy of study in FW species other than zebrafish in the future.

## 6. K+ channel-mediated K+ secretion

Unlike Na^+^, Cl^-^, and Ca^2+^, which are absorbed by hyperosmoregulatory zebrafish (see above), K^+^ was recently suggested to be secreted through a K^+^ channel, Kir1.1, an ortholog of the mammalian renal outer medullary K^+^ channel (ROMK) (Abbas et al., 2011[1]). Kir1.1 mRNA is expressed in a specific group of ionocytes expressing ATP1A1a.4 (Abbas et al. 2011[1]), distinct from other ionocyte types (HR, NCC, and NaR ionocytes, see above); Kir1.1 loss-of-function was found to induce transient tachycardia followed by bradycardia (Abbas et al., 2011[1]), effects similar to those observed in hyperkalemia in human (Kahloon et al., 2005[88]). These Kir1.1-expressing ionocytes were accordingly proposed to be K^+^-secreting (KS) cells (Abbas et al., 2011[1]), despite the lack of direct evidence for K^+^ currents in KS cells. Recent studies used a novel technique to insolubilize and visualize K^+^ excreted from a group of ROMK- and NHE3-labeled gill ionocytes in tilapia; furthermore, gill ROMK expression was found to be stimulated in tilapia acclimated to either high-K^+^ SW or high-K^+^ FW as compared with the respective SW or FW control (Furukawa et al., 2014[45], 2012[46]). ROMK-mediated K^+^ secretion appears to occur in fish under hypoosmotic FW and hyperosmotic SW. The physiological significance, particularly in FW fish like zebrafish, is a challenge issue that remains to be explored. Further studies are awaited to determine if K^+^ secretion is required not only for body fluid K^+^ homeostasis, but also to drive the relevant transporters for absorption or transport of other ions, as observed for mammalian kidneys (Hibino et al., 2010[61]; Welling and Ho, 2009[189]).

## 7. Hormonal control and functional regulation

**Figure 3:**
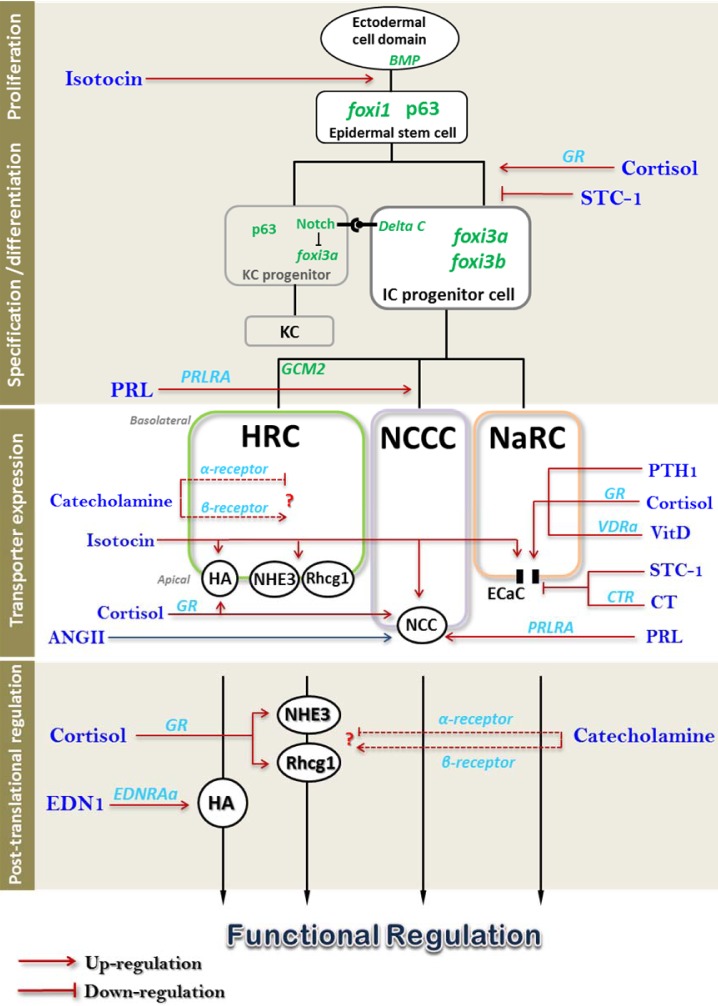
Schematic of hormone actions on the various aspects of ion regulatory processes. Different hormones differentially affect ionocyte number (cell proliferation, specification, and differentiation), transporter abundance (expression of transporter), and activity (post-translational regulation). Dashed lines and the question mark indicate that the transporters regulated by specific hormone are yet to be identified. Ionocyte specification and differentiation are presented as a simplified depiction modified from Hwang and Chou (2013); for details, refer to the text. For the ionocytes and related transporters, refer to Fig.1. IC, ionocyte; KC, keratinocyte; STC-1, stanniocalcin-1; PRL, prolactin; PRLRA, prolactin receptor A; PTH1, parathyroid hormone 1; VitD, vitamin D; VDRa, vitamin D receptor A; CT, calcitonin; CTR, calcitonin receptor; ANGII, angiotensin II; EDN1, endothelin-1; EDNRAa, endothelin receptor Aa.

As the primary link between environmental cues and physiological responses, the neuroendocrine system is a critical part of osmoregulatory mechanisms. As described above, the molecular/cellular models of ion regulation have been established more comprehensively and convincingly in zebrafish than in other traditional model species (Chang et al., 2009[17]; Hwang and Chou, 2013[78]; Hwang and Perry, 2010[82]). This makes zebrafish an attractive and competent model with which to study the hormonal control of fish ion and acid-base transport processes, as the actions of hormones can be precisely linked to effectors responsible for specific ion transport. In addition, the availability of complete genome sequences enables the ready identification of receptor isoforms in zebrafish; this is particularly important for endocrine research, because the different isoforms may mediate distinct physiological functions. Furthermore, the suitability of zebrafish for gene overexpression and knockdown (unlike more traditional model species, which are usually treated with heterologous hormones and inhibitors) provides a powerful approach to alter homologous/endogenous levels of hormones or receptors. By exploiting all of these advantages, many studies regarding hormonal action on zebrafish ion regulation have been published, which are summarized in previous reviews (Hwang and Chou, 2013[78]; Kwong et al., 2014[107]). Here, we will build on the existing reviews and incorporate new data with discussions of contentious issues, to provide an updated overview of endocrine control of ion regulation in zebrafish (Figure 3(Fig. 3)).

### 7.1 Hormonal control of Ca^2+^ uptake 

Cortisol plays an important role in regulating a wide range of physiological processes in fish, including metabolism, immune responses, growth, reproduction, and osmoregulation (Mommsen et al., 1999[133]). Because early studies failed to detect significant synthesis of aldosterone (Colombo et al., 1972[25]), it was traditionally assumed that cortisol functions as both a glucocorticoid and a mineralocorticoid through the glucocorticoid receptor (GR) in teleosts. With the discovery of a MR gene in rainbow trout (Colombe et al., 2000[24]), a more complicated mechanism is emerging for cortisol signaling in fish. Unlike its role in mammals as a hypocalcemic hormone (Patschan et al., 2001[140]), cortisol was found to act as a stimulator for Ca^2+^ uptake in fish. It has been reported that low Ca^2+^ water can enhance plasma cortisol levels (Flik and Perry, 1989[41]), and exogenous cortisol up-regulates branchial expression of ECaC mRNA and protein in rainbow trout (Shahsavarani and Perry, 2006[157]). Studies in zebrafish reinforced these findings by further investigating the underlying molecular/cellular mechanisms. Lin et al. (2011[118]) reported that low Ca^2+^ environments increase the expression of 11β-hydroxylase (the enzyme that catalyzes the last step of cortisol synthesis), and cortisol treatment increases mRNA expression of ECaC, but not that of PMCA2 or NCX1b, in zebrafish. Moreover, GR MO, but not MR MO, abolishes the enhancement of ECaC expression and Ca^2+^ uptake upon cortisol treatment (Lin et al., 2011[118]), providing convincing molecular physiological evidence, rather than the debating pharmacological ones (Kiilerich et al., 2007[91], 2011[92]; McCormick et al., 2008[132]; Takahashi and Sakamoto, 2013[168]), that the action of cortisol on ionocyte function is predominantly mediated by signaling through GR. Although there were some conflicting results from immunocytochemistry with heterologous antibodies (Cruz et al., 2013[32]; Kumai et al., 2012[102]), *in situ* hybridization with a specific GR RNA probe demonstrated the presence of GR mRNA in NaR ionocytes and other types of cells in the zebrafish gill (Lin et al., 2011[118]), suggesting that cortisol exerts direct hypercalcemic activity on ionocytes. On the other hand, cortisol may also have indirect effects on Ca^2+^ uptake through other mediators. In zebrafish, cortisol-GR signaling was also demonstrated to regulate the expression of the receptor and synthesizing enzyme of vitamin D (Lin et al., 2012[117]), a well-known hypercalcemic hormone in both mammal and fish. Additionally, cortisol treatment causes down-regulation of STC-1 expression (Kumai et al., 2015[100]), suggesting that cortisol may also regulate Ca^2+^ uptake through the hormone-counterbalancing mechanism. 

Bioactive vitamin D (VitD) is a well-documented hormone regulating Ca^2+^ uptake in mammals. Complexes of vitamin D_3_ and the vitamin D receptor (VDR) can up-regulate mammalian ECaC translation by binding to the VDR responsive element (VDRE) of ECaC, thereby enhancing Ca^2+^ absorption (Hoenderop et al., 2005[68]). The hypercalcemic action of VitD is conserved in teleosts, in which the bioactive form of Vit-D, 1α,25(OH)2D3, has been demonstrated to be produced by renal tissues and the liver (Bailly du Bois et al., 1988[5]; Hayes et al., 1986[58]; Takeuchi et al., 1991[171]), where it exerts a conserved role in the stimulation of Ca**^+^** uptake (Sundell et al., 1993[164]; Swarup et al., 1992[166]). In zebrafish embryos, application of exogenous VitD significantly stimulated ECaC expression, but had no effects on PMCA2 and NCX1b expression (Lin et al., 2012[117]). In addition, VitD treatment also exerted negative and positive effects on expression of zebrafish CYP27B1 (an enzyme that converts the precursor to bioactive vitamin D) and CYP24A1 (an enzyme that catalyzes the degradation of Vitamin D), respectively, reflecting a feedback mechanism in homeostasis of vitamin D levels as found in mammals. (Lin et al., 2012[117]). Teleosts possess two VDR paralogs (Sundell et al., 1993[164]), both of which were identified in zebrafish (Craig et al., 2008[28]). Although *in vitro* studies suggested a divergent function of the two teleost VDRs (Howarth et al., 2008[73]), it was unclear as to whether the two paralogs have similar or diverse functions on Ca^2+^ regulation *in vivo* until the recent zebrafish study. Loss-of-function analyses of the two receptors revealed that VitD-mediated enhancement of ECaC expression/function is mediated through VDRa alone, and this finding was reinforced by observed co-localization of VDRa mRNA in zebrafish NaRC cells (Lin et al., 2012[117]). In addition, transcription of the genes encoding VDRa, CYP27A1 and CYP27B1, was stimulated by exogenous cortisol, but suppressed by knockdown of the GR (but not the MR), indicating that cortisol-enhanced ECaC expression and Ca^2+^uptake may be achieved through regulating the metabolism and signaling of VitD in zebrafish (Lin et al., 2011[118]). 

Parathyroid hormone (PTH) is expressed by and secreted from the parathyroid gland in higher vertebrates. PTH functions as a hypercalcemic hormone, and can stimulate renal Ca^2+^ absorption through modulating expression of Ca^2+^-transporter in mammals (Hoenderop et al., 2005[68]; van Abel et al., 2005[180]). Although fish apparently lack an equivalent of the parathyroid gland, two fish paralogs of PTH (PTH1 and PTH2) were identified (Gensure et al., 2004[48]); expression of these genes has been detected in several zebrafish tissues, including the gills, brain, and muscle (Lin et al., 2014[116]). Several lines of evidence suggest that PTH is also involved in stimulation of Ca^2+^ uptake in fish, as observed in mammals. PTH1 expression in zebrafish was inhibited by maintenance in diluted FW supplemented with additional Ca^2+^ (Hoshijima and Hirose, 2007[72]), while PTH1 expression in zebrafish embryos was stimulated by low Ca^2+^ FW (Kwong et al., 2014[106]; Lin et al., 2014[116]). Additionally, the serum Ca^2+^ level of goldfish was enhanced following injection of heterologous PTH1 (Suzuki et al., 2011[165]), and this effect was reconfirmed in zebrafish (Lin et al., 2014[116]). Overexpression of homologous PTH1 was shown to stimulate whole-body Ca^2+^ content and ECaC expression in zebrafish embryos, while PTH2 had no such effects (Lin et al., 2014[116]); these findings provide molecular physiological evidence for the different, but not redundant, roles of the two paralogs in fish Ca^2+^ homeostasis. Three PTH receptors have been identified in zebrafish and other teleost species (Guerreiro et al., 2007[53]). Zebrafish PTH1R and PTH3R were found to be activated by PTH1 *in vitro* (Gensure et al., 2004[48]), but evidence for their roles in mediating the effect of PTH1 on Ca^2+^ uptake, as well as their localization in specific types of ionocyte (e.g. Ca^2+^-absorbing NaR ionocytes), remain lacking. In the higher vertebrates, Ca^2+ ^sensing receptor (CaSR) is expressed in the parathyroid gland, where it senses serum Ca^2+^ levels to modulate PTH secretion (Hoenderop et al., 2005[68]). CaSR expression was also identified in PTH1-expressing tissues of zebrafish, and CaSR morphants exhibited elevated PTH1 expression, suggesting that CaSR signaling negatively regulates PTH expression (Lin et al., 2014[116]). Such CaSR-mediated action has been shown to play an important role in the overall regulation of Ca^2+^ homeostasis (please refer to Section 7.4).

In addition to calciotropic hormones, hypocalcemic hormones are also involved in the regulation of Ca^2+^ uptake in fish, as in mammals. Stanniocalcin-1 (STC-1) is secreted from the corpuscles of Stannius (CS), a specific, small endocrine gland, which is attached to the kidneys in bony fish (Wagner et al., 1986[184]). STC-1 has been shown to function as a hypocalcemic hormone in fish, based on Ca^2+^ challenge experiments (Tseng et al., 2009[178]; Wagner et al., 1998[183]), surgical removal of the CS (Hanssen et al., 1989[56]), injection with CS extracts or STC-1 (Lafeber and Perry, 1988[109]), and loss-/gain-of-function experiments (Tseng et al., 2009[178]). In zebrafish embryos, STC-1 was found to reduce Ca^2+ ^uptake in response to excess Ca^2+ ^in the body by negatively regulating expression of ECaC, but not that of PMCA2 or NCX1b (Tseng et al., 2009[178]). To date, no STC receptor has been cloned/identified; however, an *in situ* ligand binding approach was used to show that the STC-1 binding site is located in the branchial ionocytes of rainbow trout (Richards et al., 2012[151]), implying that gill ionocytes may be a potent target of STC-1 action. As regards the regulation of STC-1, macrophage-stimulating protein (Msp) was reported to be a novel Ca^2+^ homeostasis modulator that may act upstream of zebrafish STC-1 (Huitema et al., 2012[76]). *In situ* hybridization revealed that the receptor for Msp, Ron, is specifically expressed in the CS. Ron knockdown morphants and msp mutant embryos presented with identical phenotypes, which could be rescued by excess Ca^2+^; the msp mutant embryos had higher levels of STC-1, ECaC, PTH1, and PTH2 expression and elevated body Ca^2+^ content as compared to wild-type embryos (Huitema et al., 2012[76]). These results suggest that the Msp/Ron signaling pathway controls Ca^2+^homeostasis, most likely through interactions with other hormones, such as STC-1 and PTH1. On the other hand, CaSR, which was found to be expressed in the CS, was proposed to be involved in the regulation of STC-1 secretion and expression in the CS (Greenwood et al., 2009[52]; Lin et al., 2014[116]). In zebrafish CaSR morphants, STC-1 expression was down-regulated, suggesting a stimulatory action of CaSR signaling on STC-1(Lin et al., 2014[116]).

Calcitonin (CT) is a small peptide produced by the parafollicular C cells of the thyroid gland in mammals. In teleosts, CT is majorly synthesized in the ultimobranchial gland (UBG) (Hazon and Balment, 1998[59]; Wendelaar Bonga, 1993[190]). CT is generally thought to be a hypocalcemic hormone in both fish and mammals (Azria, 1989[4]; Evans et al., 2005[39]; Wagner et al., 1997[185]). Injection of homologous or heterologous CT was shown to cause hypocalcemic effects in a variety of species, including stingray (Sasayama et al., 1992[154]), carp (Chakrabarti and Mukherjee, 1993[15]), and goldfish (Sasayama et al., 1992[154]). Treatment of zebrafish embryos with high-Ca^2+^ FW not only causes the aforementioned down-regulation of ECaC expression, but also up-regulated expression of CT and its receptor (CTR) (Lafont et al., 2011[110]). ECaC expression in zebrafish embryos is stimulated by knockdown of CT and inhibited by overexpression of CT, supporting an anti-calciotropic role for CT (Lafont et al., 2011[110]). However, CT was also suggested to exert biphasic effects in zebrafish. Overexpression of CT consistently suppressed ECaC and stimulated STC-1 expression in zebrafish embryos (from 30 hours to 4 days post fertilization(dpf)), but this effect was accompanied with up-regulation of several hypercalcemic factors, including PMCA2, NCX1, PTH receptors (PTH1R, PTH2R, and PTH3R), and VDR at 4 dpf (Lafont et al., 2011[110]). This result shows that CT-mediated suppression of Ca^2+^ uptake results in short-term hypocalcemia, and this may result in activation of hypercalcemic signaling to compensate for the Ca^2+^ imbalance through induction of hypercalcemic hormones and other Ca^2+^ transporters. As such, the findings from zebrafish indicate that ECaC is the primary target of CT-mediated hypocalcemic regulation, and also highlight the importance of hormone interactions on Ca^2+^ homeostasis.

### 7.2 Hormonal control of Na^+^/Cl^-^ uptake

Cortisol has long been recognized as a switch hitter to regulate hydromineral balance in both SW and FW environments (Evans et al., 2005[39]; McCormick, 2001[130]; Takahashi and Sakamoto, 2013[168]; Takei et al., 2014[169]). In zebrafish, cortisol was also found to play roles in regulating Na^+^ uptake, and such activity appears to be mediated by GR, but not MR (according to pharmacological experiments) (Kumai et al., 2012[102]). Such cortisol-GR-mediated activation of Na^+^ uptake was suggested to contribute to the tolerance of zebrafish to acidic environments, because low pH-induced compensation of Na^+^ uptake in this species was blocked by GR knockdown (Kumai et al., 2012[102]).

 Increasing evidence suggests that cortisol can regulate Na^+^ balance by suppressing ion loss, primarily through altering epithelial permeability. Exogenous cortisol treatment significantly increased mRNA and protein expression of the epithelial tight junction (TJ) proteins (claudin-a and -b), which was associated with a reduction in paracellular permeability upon acid challenge (Kwong and Perry, 2013[108]). Diffusive Na^+^ loss and reduction of whole-body Na^+^ content following acute acid exposure were mitigated by cortisol treatment (Kwong and Perry, 2013[108]). Such activity may be achieved via GR, because knockdown of GR abrogated the effects of cortisol on paracellular permeability, and GR morphants exhibited a more pronounced increase in paracellular permeability (and a greater diffusive loss of Na^+^) than control fish following acid exposure (Kwong and Perry, 2013[108]). These findings suggest that cortisol-GR-mediated regulation of permeability contributes to the acid tolerance of zebrafish by promoting Na^+^ retention, thus attenuating the disturbance of Na^+^ upon acid exposure. Although the role of cortisol in Na^+^ uptake under harsh environments is widely accepted, some debates remain. The role of cortisol-GR signaling under normal conditions is questionable at present. GR knockdown did not affect Na^+^ uptake in zebrafish reared in normal FW (Kumai et al., 2014[99], 2012[102]), conflicting with the studies by Cruz et al. (2013[32]) and Nesan and Vijayan (2013[137]), which demonstrated that GR morpholinos suppress the differentiation of HR cells and reduce Gcm2 expression, respectively, predicting a resultant impairment in HRC-mediated function. Additionally, the observation that cortisol treatment does not stimulate the expression of NHE3b and Rhcg1 (the most important transporters associated with Na^+^ uptake in HR cells) (Kumai et al., 2012[102]) is also inconsistent with the findings of Cruz et al. (2012[30]) that cortisol exposure increased HR cell number (predicting an enhancement of NHE3b expression). This discrepancy demands resolution in the future.

The pituitary hormone prolactin has long been shown to be a FW-adaption hormone, but the regulatory mechanisms by which it affects specific ion transport are still not well-understood (Breves et al., 2014[12]; Manzon, 2002[126]; Sakamoto and McCormick, 2006[152]). Previous studies of tilapia suggested that prolactin plays an important role in the regulation of NCC in FW ionocytes: transfer of hypophysectomized tilapia to FW failed to induce mRNA expression of NCC2 in the gills, but this defect was fully rescued by injecting heterologous prolactin into the fish; this implies that prolactin is responsible for inducing NCC2 expression, and possibly also for causing the differentiation of NCC-expressing ionocytes (Breves et al., 2010[14]). Recently, the action of PRL on NCC was further reinforced in zebrafish. Intraperitoneal injection of ovine PRL into adult zebrafish significantly stimulated the gene expression of NCC2b and one of the prolactin receptors, PRLRA, in the gills within 48 h, but did not affect that of NHE3b or ECaC (Breves et al., 2013[13]). Additionally, treatment of cultured gill filaments with oPRL increased the transcript level of NCC2b in a dose-dependent manner, and this effect was blocked by a human PRL receptor antagonist (D1-9-G129R-hPRL) (Breves et al., 2013[13]). These findings from both *in vivo* and i*n vitro* experiments demonstrated that PRL is necessary and sufficient to regulate the expression of NCC in zebrafish gills. PRL knockdown resulted in a reduction of NCC-expressing cells in zebrafish embryo (Breves et al., 2014[12]), suggesting that PRL may regulate levels of NCC through control of both NCC transcription and ionocyte differentiation. Given that NCC is a key player for Cl^-^ uptake (as well as Na^+^ uptake) in zebrafish, these findings provided new insights into the hormonal mechanism of Cl^-^ regulation. Nevertheless, whether prolactin affects other types of ionocytes and/or transporters remains to be seen. In a study by Breves et al. (2014[12]), it was reported that PRL knockdown merely reduced the number of NCC-expressing cells (and thus the expression of NCC2b), while NHE3b- and ECaC-expressing cells were not affected. On the other hand, it should be noted that in another study the decreased expression of NCC2b (by MO knockdown) resulted in a compensatory increase of HR cell (i.e., NHE3-expressing cell) number (Chang et al., 2013[19]). This inconsistency will require further investigation to clarify.

The role of the renin-angiotensin II system (RAS) in promoting salt reabsorption in the mammalian kidney is well documented (Crowley and Coffman, 2012[29]). While RAS is also known to be involved in the control of body fluid volume and blood pressure in fish (Bernier et al., 1999[7]; Takei et al., 2014[169]; Takei and Tsuchida, 2000[170]; Tierney et al., 1995[176]), its role in ion regulation of FW fish remained largely unexplored until recently. A previous study by Hoshijima and Hirose (2007[67]) reported an increase of renin mRNA expression in zebrafish embryos acclimated to ion-poor FW, suggesting an involvement of RAS in the regulation of salt absorption. Recently, the contribution of RAS to Na^+^uptake was investigated in embryonic zebrafish. The level of whole-body ANG-II was significantly increased after acute exposure to one of two conditions that impede Na^+^ uptake, acidic (pH 4) or ion-poor FW (Kumai et al., 2014[99]), suggesting rapid activation of the RAS in zebrafish. Acute exposure to acidic water or ion-poor water resulted in a significant increase in Na^+^uptake, and this enhancement could be partially blocked using an ANG-II receptor antagonist (Kumai et al., 2014[99]). In support of these results, knockdown of renin prevented stimulation of Na^+^uptake following acute exposure to acidic or ion-poor water (Kumai et al., 2014[99]). The action of RAS under acute challenge seems to be cortisol-independent, because RU486 treatment or GR knockdown did not affect stimulation of Na^+^uptake during acute exposure to acidic or ion-poor conditions (Kumai et al., 2014[99]). The exact identity of the Na^+^ transporter that is regulated by ANG-II remains unclear. The elevation of Na^+^uptake following chronic (24 h) waterborne treatment with ANG-II was accompanied by increased NCC2b expression (Kumai et al., 2014[99]), implying that the NCC-mediated pathway is involved in RAS action. As such, ANG-II may affect not only Na^+^, but also Cl^-^ handling in zebrafish. However, more evidence is required to verify these findings, e.g., identification of the target transporters and localization of ANG-II receptors.

Neurohormones, such as catecholamines, also contribute to Na^+^ uptake in zebrafish. Catecholamines released from either nerves or chromaffin cells (Reid et al., 1998[150]) have long been suspected to play an important role in ionoregulation in FW fish through their actions on α- and β-adrenergic receptors (for details, please refer to the following review: Evans et al., 2005[39]). The effects of catecholamine on fish ion regulation were verified in zebrafish through pharmacological and knockdown approaches. Pharmacological screening was performed to identify α- and β-adrenergic receptors with inhibitory and stimulatory roles, respectively, on Na^+^ uptake in zebrafish embryos (Kumai et al., 2012[105]). Knocking down β receptor with specific MOs impaired Na^+^ uptake under acidic or ion poor environments, indicating that activation of β-adrenergic receptors contributes to the increase in Na^+^ uptake upon acid challenge (Kumai et al., 2012[105]). Although there was no attempt to identify the specific Na^+^ transporter(s) activated by β-adrenergic receptor stimulation, it was found that HR cells can be labeled with fluorescent propranolol (a β receptor antagonist) and zn-12 (a genetic neuronal marker) (Kumai et al., 2012[105]), implying that transporters located in HR cells may play a critical role in β-adrenergic signaling-mediated stimulation of Na^+^ uptake.

β-adrenergic receptors, once stimulated, activate transmembrane adenylyl cyclase (tAC), which in turn triggers the downstream signaling pathways mediated by 3′,5′-cyclic adenosine monophosphate (cAMP). Treating larval zebrafish with forskolin (tAC activator) or 8-bromo-cAMP significantly increased Na^+^ uptake (Kumai et al., 2014[101]). Acute exposure to acidic water resulted in both enhanced Na^+^ uptake and an elevation in whole body cAMP levels, which was attenuated by inhibiting PKA; this finding suggests that the cAMP-PKA pathway mediates Na^+^ uptake regulation (Kumai et al., 2014[101]). The control of intracellular signaling by cAMP may regulate multiple Na^+^ transporters, including NHE3b and possibly NCC2b (Kumai et al., 2014[101]). However, β-Adrenergic receptors do not appear to be localized to NCC cells (Kumai et al., 2012[105]), and thus cAMP may also mediate signals from hormones other than catecholamines.

While most studies have focused on the hormonal mechanisms that contribute to stimulating Na^+^ uptake, α-adrenergic regulation of Na^+^ uptake suppression hinted at a possible pathway that negatively controls Na^+^ transport. Recently, hydrogen sulfide (H_2_S), a gas molecule known to play an important role in cardiorespiratory control/oxygen sensing, was discovered to be an inhibitory regulator of Na^+^ uptake in developing zebrafish (Porteus et al., 2014[147]). Waterborne treatment with Na_2_S (a chemical known to generate H_2_S) significantly reduced Na^+^ uptake in acid-treated zebrafish embryos, but did not affect uptake in Gcm2 morphants (Porteus et al., 2014[147]), which lack HR ionocytes and absorb Na^+^ predominantly via NCC cells (Chang et al., 2013[19]); collectively, these findings suggest that Na^+^ uptake via NHE3b, but not NCC2b, is regulated by H_2_S (Porteus et al., 2014[147]). Although HR ionocytes have been suggested to endogenously produce H_2_S for the inhibition of Na^+^ uptake in zebrafish (Porteus et al., 2014[147]), H_2_S was also shown to stimulate catecholamine secretion in rainbow trout (Perry et al., 2009[142]), implying that the H_2_S-α-adrenergic pathway may mediate negative regulation of Na^+^ uptake in fish. This is an interesting issue that deserves further investigation.

### 7.3 Hormonal control of acid secretion

At present, our understanding of the hormonal control of acid-base regulation in fish is fragmentary. Most studies on this issue have focused on ion transport regulation to compensate for the disruption in ion balance under acidic stress. Several hormones, including cortisol (Kumai et al., 2012[102]), prolactin (Flik et al., 1989[42]), somatolactin (Kakizawa et al., 1996[89]), endothelin 1 (EDN1) (Guh et al., 2014[54]), and angiotensin II (ANG-II) (Kumai et al., 2014[99]), have been reported to be induced after exposure to acidic water. With the exceptions of cortisol and EDN1, the involvement of these hormones in H^+^ transport regulation remains unclear. 

Cortisol has long been reported to participate in regulation of H^+^ secretion in FW fish. H^+^ -ATPase activity is stimulated in gill homogenates of rainbow trout following exogenous cortisol treatment (Lin and Randall, 1993[120]). Moreover, the whole body cortisol level was significantly elevated in zebrafish embryos after exposure to acidic water (Kumai et al., 2014[99]). Our recent SIET data demonstrated that cortisol exposure stimulates H^+^ secretion in the skin of embryonic zebrafish (Figure 2B(Fig. 2)) (Lin et al., unpublished data). The underlying mechanism of cortisol action on H^+^ secretion remains unclear. However, Cruz et al. (2012[30], 2013[32]) reported that cortisol treatment increased the number of ionocytes (including HR cells) through GR-mediated stimulation of Foxi3a, suggesting that cortisol affects H^+^ secretion, at least partly, through regulating the proliferation/differentiation of HR ionocytes. 

EDN is a family of three 21 amino acid peptides (EDN1, -2, and -3), which function in a variety of physiological processes, including regulation of vascular tone and renal ion/water transport (Kohan et al., 2011[95]). EDN1 is an important regulator of H^+^ secretion in mammalian kidney (Alpern and Preisig, 2004[2]; Wesson, 2006[192], 2011[191]). In zebrafish, expression of EDN1 and one of its receptors, EDNRAa, was stimulated in adult gills and whole embryo following acclimation to acidic water (Guh et al., 2014[54]). EDN1 overexpression enhanced H^+^ secretion (as detected by SIET) in the embryonic skin, while EDNRAa loss-of-function significantly decreased EDN1- or low pH-induced H^+^ secretion (Guh et al., 2014[54]). EDN1 seems to function through modulating H^+^-ATPase, because EDN1-enhanced H^+^ secretion was impaired by a H^+^-ATPase inhibitor, bafilomycin A1 (Guh et al., 2014[54]). EDN1 does not appear to affect H^+^ secretion through either altering the abundance of H^+^-ATPase or affecting the cell differentiation of HR ionocytes, based on Western blot and immunocytochemistry experiments in EDNRAa morphants (Guh et al., 2014[54]). This suggests that EDN1 may influence H^+^ secretion through regulating post-translational process(es) in an acute manner soon after acid challenge. Supporting this notion, our preliminary experiments have shown that expression of EDN1 is stimulated 2 h after acidic (pH 4) FW treatment (Guh et al., unpublished data).

### 7.4 “Crosstalk” of hormone actions

**Figure 4:**
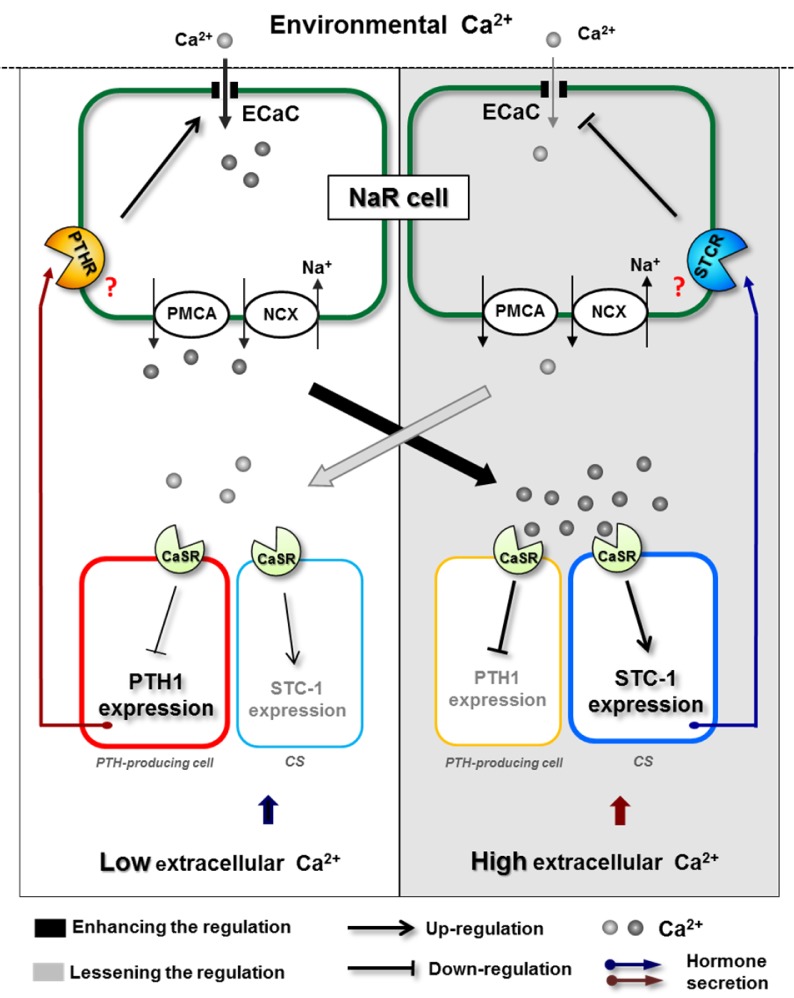
Proposed model of Ca^2+^ homeostasis through the counterbalance of hypercalcemic and hypocalcemic hormones. High extracellular Ca^2+^ (right panel) activates CaSR to stimulate and inhibit the expression of STC1 and PTH, respectively. The resultant hypocalcemic effect suppresses Ca^2+^ uptake from ECaC and thereby reduces the extracellular Ca^2+^ level, subsequently relieving CaSR signaling (left panel) as a feedback mechanism. For details, refer to the text. NaR cell, Na^+^-K^+^-ATPase rich cell; PTHR, putative PTH1 receptor; STCR, putative STC-1 receptor.

The accumulation of findings addressing ion regulation by various hormones has underscored the importance of interaction or counterbalancing among different hormones. A very important case in fish ion regulation is the synergy between growth hormone (GH) and cortisol to improve the salinity tolerance of fish (McCormick, 2001[130]). In several salmonid species, injection of both GH and cortisol increased gill Na^+^- K**^+^-**ATPase activity and salinity tolerance to a greater extent than that caused by either hormone alone (Madsen, 1990[125]; McCormick, 1996[129]). This synergistic action was suggested to achieve through the mechanism that GH increases the abundance of gill cortisol receptors (Shrimpton et al., 1995[161]; Shrimpton and McCormick, 1998[162]). In addition, GH was also found to increase the sensitivity of interrenal tissue to ACTH, and thereby stimulate secretion of cortisol (Young, 1988[202]). In zebrafish, a representative example of hormone interactions in the context of ion handling is the counterbalanced regulation of hypercalcemic and hypocalcemic hormones on Ca^2+^ homeostasis (Figure 4(Fig. 4)). As mentioned above, calciotropic PTH1 can enhance Ca^2+^ content and ECaC expression in zebrafish (Lin et al., 2014[116]), while hypocalcemic STC-1 possesses an inhibitory effect (Tseng et al., 2009[178]). These two hormones were found to mutually stimulate each other's expression (Lin et al., 2014[116]). This mutual effect was proposed to converge on the mediation of CaSR. CaSR, as a Ca^2+^sensor, was demonstrated to inhibit PTH1 expression and to stimulate STC-1 expression in a time-dependent manner (Lin et al., 2014[116]). Overall, under a low Ca^2+^ environment, PTH1 is induced and enhances compensatory Ca^2+^ uptake in NaR ionocytes. Following the accumulation of extracellular Ca^2+^, activation of CaSR initiates downstream signaling to up-regulate STC-1 expression in the CS and concomitantly down-regulate PTH1 expression in the PTH-producing cell, thereby suppressing Ca^2+^ absorption in the ionocyte. The suppression of Ca^2+^ uptake and the resultant reduction of systemic Ca^2+^ relieves CaSR signaling, and consequently functions as a feedback mechanism to limit hypocalcemic activity. 

The awareness of crosstalk among hormones coordinating body fluid Ca^2+^ homeostasis emphasizes that data addressing hormone actions need to be interpreted with caution. Recently, Kumai et al. (2014[101]) reported that Gcm2, a transcription factor related to HR cell differentiation and the development of pharyngeal cartilage, plays a critical role in zebrafish Ca^2+^ homeostasis. Gcm2 was revealed to positively affect the number of ECaC-expressing cells (i.e., NaR ionocytes) as well as Ca^2+^ uptake in embryonic zebrafish, and this effect was linked to the regulatory network by which Gcm2 transactivates CaSR and mediates the hypercalcemic action of cortisol. A positive correlation between Ca^2+^ transport function and the expression of Gcm2 is both plausible and attractive. However, certain conflicts, such as the experiments that acidic FW stimulates Gcm2 expression with a concomitant decrease in zebrafish Ca^2+^ content, suggest that the effects of environmental acidification or Gcm2 knockdown on zebrafish acid-base regulation might be considered to be primary factors with which to interpret correlated data regarding transport of Ca^2+^ (or other ions), which probably reflect a secondary or compensatory response. More detailed observations of temporal changes of related parameters are necessary to accurately elucidate the proposed pathways. Moreover, Gcm2 is specifically expressed in HR ionocyte types, but not in ECaC-expressing cells or other ionocytes (Chang et al., 2009[17]; Esaki et al., 2009[36]). Whether Gcm2 is co-localized with CaSR in cells that secrete the related hormones is not determined. Therefore, it is not clear how Gcm2 interacts with CaSR or mediates cortisol activity (proposed to function via ECaC cells).

The crosstalk among endocrine systems is also important for the coordination of ion transport processes across acute and acclimation phases. Hormones that are activated in the acute phase may be important signals for release of hormones in the acclimation phase (McCormick and Bradshaw, 2006[131]; Takei et al., 2014[169]). As noted above, ANG-II was shown to be induced by acute acidic exposure (Kumai et al., 2014[99]), and may serve as a fast-acting hormone in the regulation of Na^+^ handling during this phase. Additionally, cortisol was thought to participate in chronic Na^+^ uptake (Kumai et al., 2012[102]). Interestingly, ANG-II was found to enhance the secretion of cortisol in several fish species, including eels, trout, and flounder (for details, please refer to the review by Takei et al., 2014[169]). In this context, ANG-II may act as a signal for cortisol production, resulting in chronic regulation of Na^+^ uptake for both fine-tuning and long-term regulation. This issue awaits further exploration. 

### 7.5 Hormonal control of ionocyte differentiation

Fish have to regulate their ion transport functions to maintain body fluid ionic/osmotic homeostasis in a harsh environment as described above. Such regulation is achieved though not only adjustments at the activity level of transporters, but also cytogenetic effects on ionocytes. Regulation at the cytogenetic level is important for long-term acclimatory processes, and is thus tightly controlled by hormones (please see the following reviews: Evans et al., 2005[39]; McCormick, 2001[130]; Takei et al., 2014[169]). Increased numbers of gill ionocytes in trout, guppy, and eel during acclimation to salinity changes was thought to be derived from enhanced mitotic activity and turnover rate, based on early cell biological experiments (Conte and Lin, 1967[26]; Chretien and Pisam, 1986[23]; Wong and Chan, 1999[194]). Recent use of immunocytochemistry to make sequential *in vivo* observations of tilapia demonstrated that the expression and function of transporters in most pre-existing ionocytes are modified, without any change in cell turnover during 1-3 d of salinity or low-Cl^-^ artificial FW treatment (Choi et al., 2010[20]; Hiroi et al., 1999[63]; Inokuchi and Kaneko, 2012[86]; Lin et al., 2004[115]). Whether the increase in ionocytes originates from the renewal of new cells or the transformation from pre-existing cells is seemingly dependent on species and/or the type or intensity of environmental stresses. Integration of earlier physiological and pharmacological data resulted in a pioneering model to address the hormonal effects on the proliferation and differentiation of gill ionocytes during acclimation to SW or FW (McCormick, 2001[130]; Sakamoto and McCormick, 2006[152]); in the gills, proliferation and differentiation of SW-type ionocytes are regulated by GH, IGF-I, and cortisol, whereas FW-type cells are regulated by prolactin and cortisol. The model previously lacked supporting molecular evidence because the identities of the stem cells, precursors, or differentiating ionocytes in fish gill epithelium were unknown until recent studies in zebrafish (Chang and Hwang, 2011[18]; Hwang and Chou, 2013[78]). The first model encompassing molecular pathways of proliferation, specification, and differentiation of fish gill/skin ionocytes was established in zebrafish (Hsiao et al., 2007[74]; Hwang and Lee, 2007[79]) (Figure 3(Fig. 3)) by loss- and gain-of-function and transgenic fish approaches (Chang et al., 2009[17]; Esaki et al., 2007[35], 2009[36]; Hsiao et al., 2007[74]; Janicke et al., 2007[87]). The different types of ionocyte originate from non-neural ectoderm that expresses ΔNp63 (a marker of proliferating epithelial cells). Initially, two specification markers of ionocytes, forkhead box I3a (Foxi3a) and DeltaC (a ligand of Notch signaling), are expressed in certain ΔNp63-positive epidermal cells, which later become ionocyte progenitors. The progenitor cells undergo lateral inhibition through Notch signaling, and differentiate into two cell lineages: (1) ionocytes (differentiation activated by Foxi3a) and (2) keratinocytes (Notch signaling maintains this cell fate by suppressing Foxi3a expression). Thereafter, the differentiating cells further give rise to different types of ionocyte, expressing the respective sets of transporters or enzymes, through complicated networks mediated by Foxi3a/-b, Gcm2 (a transcription factor specific for differentiation of HR ionocytes), and Notch signaling. The zebrafish model arguably provides a more competent platform to precisely identify and study the actions of hormones at targeted developmental stages of fish ionocytes, during acclimation to a harsh environment (Figure 3(Fig. 3)). 

In zebrafish, acclimation to ion (Ca^2+^, Na^+^, or Cl^-^) deficient, high NH_4_^+^, or acidic FW for several days to two weeks has been reported to result in both enhanced expression of genes encoding related ion transporters (Bayaa et al., 2009[6]; Craig et al., 2007[27]; Hoshijima and Hirose, 2007[72]; Horng et al., 2009[71]; Pan et al., 2005[138]; Shih et al., 2008[158]; Wang et al., 2009[187]; Yan et al., 2007[200]) and increased numbers of specific types of ionocyte in the embryonic skin or the gills (Chang et al., 2009[17]; Horng et al., 2009b[71]; Pan et al., 2005[138]; Wang et al., 2009[187]). The molecular and cellular mechanisms of functional regulation of related ion transporters and ionocytes have been studied in detail, particularly under acclimation to acidic environments: acid acclimation induces a compensatory enhancement in acid secretion capacity, which is achieved not only by increasing the acid-secreting function of individual HR cells, but also by increasing HR cell number (Horng et al., 2009b[71]); the additional HR cells in acidic FW originate from Gcm2-mediated differentiation of both ionocyte precursor cells and newly proliferating epithelial stem cells (Chang et al., 2009[17]; Horng et al., 2009b[71]). Recent studies further explored how control of the proliferation and/or differentiation of ionocytes by isotocin, cortisol, and stanniocalcin (STC-1) affect iono-/osmoregulatory mechanisms in zebrafish. 

Oxytocin is involved in many physiological functions, and is primarily produced in neurons of the hypothalamo-neurohypophysial system in mammals (Gimpl and Fahrenholz, 2001[51]). Oxytocin regulates body fluid osmotic homeostasis by simultaneously stimulating water uptake and reducing Na^+^ reabsorption (Haanwinckel et al., 1995[55]). Fish isotocin is the homolog of mammalian oxytocin, and its role in iono-/osmoregulation was proposed based primarily on correlated data of the effects of environmental salinities on the expression or secretion of isotocin and/or its receptors in several fish species (Hyodo et al., 2004[83]; Kleszczynska et al., 2006[94]; Martos-Sitcha et al., 2014[127]; Warne et al., 1994[188]); however, neither the underlying molecular and cellular mechanisms, nor the target transporter or ionocytes, are known. In zebrafish, isotocin was recently identified to have cytogenetic effects on skin/gill iono-regulatory mechanisms (Chou et al., 2011[21]). Loss- and/or gain-of-function experiments demonstrated that body ion (Na^+^, Ca^2+^, and Cl^-^) contents, expression of related genes, and the numbers of various ionocytes are all modulated by isotocin, and the underlying mechanism involves the effects on the densities of both p63-positive stem cells and Foxi3a-expressing ionocyte progenitors (Chou et al. 2011[21]). Zebrafish isotocin increases ionocyte density through stimulation of cell proliferation/differentiation, and ultimately regulates ionocyte functions. This novel finding provided a new viewpoint of the role of isotocin in fish ion regulatory mechanisms. Oxytocin is known to be involved in cell growth and differentiation in the heart, bone, and mammary gland in mammals (Gassanov et al., 2008[47]; Sapino et al., 1993[153]; Tamma et al., 2009[172]), and therefore the findings from zebrafish provide possible clues toward the existence of similar cytogenetic actions on ion transport functions in mammalian renal tubular cells.

Cortisol has long been thought to act as both a FW- and SW-adapting hormone by interacting with prolactin and growth hormone/IGF-I, respectively (Evans et al., 2005[39]; McCormick, 2001[130]; Sakamoto and McCormick, 2006[152]; Takei et al., 2014[169]). In many fish species, cortisol appears to play a pivotal role in the ion uptake mechanism during acclimation to FW, mainly in light of the correlated data of ion fluxes, density of gill ionocytes, gill expression of cortisol receptors and ion transporters, and/or plasma cortisol level (for details, please refer to the following reviews: Takahashi and Sakamoto, 2013[168]; Takei et al., 2014[169]). Exogenous cortisol was reported to stimulate the cell size, structure, and/or density of ionocytes in different fish species acclimated to FW (Madsen, 1990[125]; McCormick, 1990[128]; Perry et al., 1992[141]). Whether these cellular changes are involved in cell proliferation and/or differentiation is still uncertain, because cortisol was generally speculated to regulate differentiation when only the cell morphology was changed, while cortisol was presumed to affect proliferation if only the density was increased (Takahashi and Sakamoto, 2013[168]; Takei et al., 2014[169]). By using p63, Foxi3a/-b, and Gcm2 as markers, cortisol treatment for as short as 14-24 h was found to increase the densities of both progenitor and mature ionocytes in embryos or isolated adult gills, but the same treatment (even for a longer period, 48 h) did not affect those of stem cells and dividing cells (examined using PH3 as a marker), indicating that cortisol acts on cell differentiation, but not on cell proliferation (Cruz et al., 2013[31], [32]). Subsequent loss-of-function experiments reinforced this conclusion and further demonstrated the pathways to be mediated by GR, but not MR (Cruz et al., 2013[31], [32]). Recent studies in medaka using a similar knockdown approach also addressed the effect of cortisol-GR2 (but not GR1 and MR) on the biogenesis of ionocytes through fine-tuning of developmental timing (Trayer et al., 2013[177]). The progress made with the convincing molecular physiological data from zebrafish and medaka clarified our long-term misinterpretation of the effects of cortisol on fish iono-/osmoregulation during acclimation to FW. 

In contrast to isotocin and cortisol, which are positive regulators, STC-1 was recently identified as a negative regulator of the transport functions of ions other than Ca^2+^, as mediated through the regulation of ionocyte differentiation in FW fish (Chou et al., 2015[22]). The hypocalcemic action of STC-1 in fish has been well-documented (refer to Section 7.1). On the other hand, STC-1 may have a broader role than classical hypocalcemic function, because rainbow trout injectted with dexamethasone exhibited increased plasma STC-1 levels and a concomitant decrease in plasma Na^+^, Cl^-^, and Ca^2+^ concentrations (Pierson et al., 2004[146]). In mammals, STC-1 is also involved in control of natriuresis and kaliuresis, in addition to Ca^2+^ homeostasis (Madsen et al., 1998[124]; Turner et al., 2010[179]; Wagner et al., 1997[186]). A recent study in zebrafish revealed that expression of STC-1 mRNA is decreased by acidic or ion-deficient (other than low Ca^2+^) FW treatments, while STC-1 overexpression impairs hyperosmotic regulation mechanisms by decreasing H^+^ secretion ability and whole body Ca^2+^, Na^+^, and Cl^- ^contents, with concomitant down-regulation of the related transporters; STC-1 was further found to suppress Foxi3a-mediated ionocyte differentiation without affecting stem cells (examined using p63 as a marker), according to loss- and gain-of-function experiments (Chou et al., 2015[22]). Indeed, STC-1-mediated modulation of proliferation and/or differentiation are associated with cellular or physiological processes in cancer cells, adipocytes, and keratinocytes in mammals (He et al., 2011[60]; Sapino et al., 1993[153]; Yeung and Wong, 2011[201]). As such, the cytogenetic effects of STC-1 on various physiological processes may have been conserved from fish to human. The findings in zebrafish not only elaborate on a long-term overlooked issue (Pierson et al., 2004[146]), but have also led us to reconsider the roles of STC-1 in fish iono-/osmoregulation mechanisms.

## 8. Conclusions and perspectives

Five types of ionocyte expressing distinct sets of ion transporter (and/or enzymes) carry out the transport functions of certain ions in zebrafish gills and skin. Convincing molecular physiological evidences have been used to build the zebrafish model of ionic and acid-base regulation mechanisms which answers many long-term unsolved questions, and also offers new insights and directions for research into related issues. Further identification, localization, and functional analysis of basolateral or other cofunctional transporters (particularly in SLC26-expressing cells and KS cells) are required to more comprehensively understand fish ion-/osmoregulatory mechanisms. Expression and function of HR- and NCC-types of ionocyte (and their major transporters, NHE3b and NCC2b) are differentially regulated in a mutually compensatory pattern, to cope with physiological or environmental stresses that disturb body fluid Na^+^ homeostasis in zebrafish. Future studies may determine if SLC26-expressing and NCC cells also exhibit such functional redundancy in terms of Cl^-^ uptake and body fluid Cl^-^ homeostasis in zebrafish and other FW fishes.

Some hormones that were previously proposed to control FW fish ion regulation have had their actions re-clarified by detailed molecular/cellular/physiological investigations into the precise pathways and target transporter/ionocytes in the zebrafish model. Isotocin, PRL, cortisol, STC-1, CT, EDN1, PTH1, VitD, catecholamines, and RAS have been demonstrated to positively or negatively regulate Na^+^/Cl^-^/Ca^2+^ uptake and acid secretion through specific receptors at different stages of ionocytes, at either the transcriptional/translational or posttranslational level. Future efforts may focus not only on the actions of a single hormone, but also on the crosstalk among hormones, which is less understood in the context of FW fish ion regulation. The mutual counterbalance or feedback actions among hormones are important for the coordination of ion transport processes that maintain body fluid ionic homeostasis in fish in harsh environments. The crosstalk between STC-1 and PTH1 through CaSR in zebrafish provides a paragon that has enabled us to explore new pathways involving other hormones.

The ionocytes in zebrafish are analogous to various renal tubular cells, in terms of their ion transporter expression and function (Hwang and Chou, 2013[78]). Furthermore, hormone systems and their possible actions are conserved between zebrafish and mammals (Lohr and Hammerschmidt, 2011[123]). As such, the knowledge obtained in zebrafish also informs studies on mammals or other animal species, thereby providing insights into related fields. However, the drawbacks of the zebrafish model should not be overlooked. While pharmacological and loss-of-function approaches are comparatively easy and efficient methods with which to assay the ion regulatory roles of hormones or receptors in zebrafish *in vivo*, the necessary reagents or morpholinos affect whole zebrafish embryos; as such, interpretations of cause and effect should be made cautiously, pending further experiments to tease apart the compensatory responses of different transporters or isoforms, and the effects of crosstalk between systemic/local or different hormones. Determining the location of hormone receptors in the effector tissue/cell and analyzing temporal changes of related parameters are prerequisite to accurately elucidate hormone actions.

## Acknowledgement

Our original research was financially supported by the grants to P. P. H. from Academia Sinica and the Ministry of Science and Technology, Taiwan, R.O.C. We extend our thanks to the Core Facilities ICOB and Taiwan Zebrafish Core Facility for the technical support during the experiments. We thank Y. C. Tung for her technical and secretarial assistance.

## Conflict of interest

The authors declare that they have no conflict of interest.
